# Surveillance of Gram-negative bacteria: impact of variation in current European laboratory reporting practice on apparent multidrug resistance prevalence in paediatric bloodstream isolates

**DOI:** 10.1007/s10096-016-2869-4

**Published:** 2016-12-26

**Authors:** J. A. Bielicki, D. A. Cromwell, A. Johnson, T. Planche, M. Sharland, for the ARPEC project

**Affiliations:** 1 Paediatric Infectious Diseases Research Group (PIDRG), Institute for Infection and Immunity, St George’s, University of London, Jenner Wing, Cranmer Terrace, London, SW17 0RE UK; 2 Department of Health Services Research and Policy, London School of Hygiene and Tropical Medicine, London, UK; 3 Department of Healthcare-Associated Infections and Antimicrobial Resistance, Centre for Infectious Disease Surveillance and Control, Public Health England, London, UK; 4 Institute for Infection and Immunity, St George’s, University of London, London, UK

## Abstract

This study evaluates whether estimated multidrug resistance (MDR) levels are dependent on the design of the surveillance system when using routine microbiological data. We used antimicrobial resistance data from the Antibiotic Resistance and Prescribing in European Children (ARPEC) project. The MDR status of bloodstream isolates of *Escherichia coli*, *Klebsiella pneumoniae* and *Pseudomonas aeruginosa* was defined using European Centre for Disease Prevention and Control (ECDC)-endorsed standardised algorithms (non-susceptible to at least one agent in three or more antibiotic classes). Assessment of MDR status was based on specified combinations of antibiotic classes reportable as part of routine surveillance activities. The agreement between MDR status and resistance to specific pathogen–antibiotic class combinations (PACCs) was assessed. Based on all available antibiotic susceptibility testing, the proportion of MDR isolates was 31% for *E. coli*, 30% for *K. pneumoniae* and 28% for *P. aeruginosa* isolates. These proportions fell to 9, 14 and 25%, respectively, when based only on classes collected by current ECDC surveillance methods. Resistance percentages for specific PACCs were lower compared with MDR percentages, except for *P. aeruginosa*. Accordingly, MDR detection based on these had low sensitivity for *E. coli* (2–41%) and *K. pneumoniae* (21–85%). Estimates of MDR percentages for Gram-negative bacteria are strongly influenced by the antibiotic classes reported. When a complete set of results requested by the algorithm is not available, inclusion of classes frequently tested as part of routine clinical care greatly improves the detection of MDR. Resistance to individual PACCs should not be considered reflective of MDR percentages in Enterobacteriaceae.

## Introduction

Bacteria resistant to multiple antibiotics have been identified as a major challenge for patient management and public health [1, 2]. Multidrug-resistant Gram-negative bacteria (MDR-GNB) are considered to be particularly worrying because the therapeutic options are limited [3, 4]. Furthermore, certain MDR-GNB, such as those producing extended-spectrum beta-lactamases or carbapenemases encoded on plasmids, are of concern due to their potential for interspecies plasmid transfer [5, 6].

Large-scale national and international surveillance is an important tool in monitoring MDR-GNB resistance trends [7]. At present, most surveillance relies on collecting results from traditional antibiotic susceptibility testing (AST) to track resistance epidemiology, including multidrug resistance (MDR) [8–10]. It is, therefore, important that the comparability of isolates identified as MDR by surveillance databases is established. Standardised algorithms for reporting isolates as MDR were proposed in 2012 by a group of international experts, but these rely on a large number of antibiotics being included in AST (Table 1) [11]. The selection of antibiotic classes for routine testing continues to be highly variable [16–19]. This potentially presents a major challenge for estimating and comparing MDR-GNB prevalence from routine data, given that individual laboratories may not test all antibiotic classes required.

**Table 1:** Summary of the sets of antibiotic classes recommended for the detection of MDR-GNB (algorithm) and available from ARPEC and EARS-Net [11, 12]. In addition, pathogen–antibiotic class combinations (PACCs) used by different surveillance networks are shown [7, 13–15]

Pathogens	*E. coli*								*K. pneumoniae*								*P. aeruginosa* ^a^							
	Sets				PACCs				Sets				PACCs				Sets				PACCs			
Antibiotic classes	MDR algorithm	ARPEC	EARS-Net	Routine	ECDC	WHO	US	UK	MDR algorithm	ARPEC	EARS-Net	Routine	ECDC	WHO	US	UK	MDR algorithm	ARPEC	EARS-Net	Routine	ECDC	WHO	US^b^	UK
Aminoglycosides	X	X	X	X				X	X	X	X	X					X	X	X	X				
Anti-MRSA cephalosporins	X								X															
Anti-pseudomonal penicillins plus beta-lactamase inhibitor	X	X		X					X	X		X					X	X	X	X				
Carbapenems	X	X	X	X	X		X	X	X	X	X	X	X	X	X	X	X	X	X	X	X	X		X
Non-extended spectrum cephalosporins (first- and second-generations)	X	X							X	X														
Extended-spectrum cephalosporins (third- and higher generations)	X	X	X	X	X	X		X	X	X	X	X	X	X	X	X	X	X	X	X				X
Cephamycins	X	X							X	X														
Fluoroquinolones	X	X	X	X		X		X	X	X	X	X					X	X	X	X				
Folate pathway inhibitors	X	X		X					X	X		X												
Glycylcyclines	X								X															
Monobactams	X	X							X	X							X	X						
Penicillins (ampicillin)	X	X	X	X																				
Penicillins plus beta-lactamase inhibitor	X	X		X					X	X		X												
Phenicols	X	X							X	X														
Phosphonic acids	X								X								X							
Polymyxins	X								X								X							
Tetracyclines	X	X							X	X														
Number of antibiotic classes included in sets used to calculate the % of MDR-GNB isolates	17	13	5	8	–	–	–	–	16	12	4	7	–	–	–	–	8	6	5	5	–	–	–	–

^a^For *P. aeruginosa*, all antibiotic classes only include antibiotics with antipseudomonal activity

^b^Note that *P. aeruginosa* is not included in the US National Healthcare Safety Network (NHSN) surveillance

The monitoring of specific pathogen–antibiotic class combinations (PACCs) can be an alternative surveillance strategy to make best use of the available routine data [7, 12–14]. Some PACCs have been suggested as being useful for MDR-GNB assessment based on the recognition of an association in resistance between different antibiotic classes [15].

Using data on neonatal and paediatric GNB isolates obtained from the Antibiotic Resistance and Prescribing in European Children (ARPEC) project, this study evaluates the degree to which estimated levels of MDR are dependent on surveillance system design when routine microbiological data are used.

## Materials and methods

### Data source

The study used data from the ARPEC project, which was co-funded by the European Commission DG Sanco through the Executive Agency for Health and Consumers [20, 21].

ARPEC collected anonymised data on antimicrobial resistance between January 2011 and December 2012 from 19 European laboratories located in 12 different countries, each processing samples for one paediatric department or hospital. ARPEC requested that participating laboratories reported AST results for isolates of a specified set of bacterial species, and that, where possible, laboratories report on specific antibiotics. These included antibiotics required for the European Antimicrobial Resistance Surveillance Network (EARS-Net) 2010 reporting protocol plus some additional antibiotic categories (Table 1) [12, 22]. The AST results for each antibiotic tested were reportable as susceptible/intermediate/resistant (S/I/R) using breakpoints defined by either:

1. European Committee on Antimicrobial Susceptibility Testing (EUCAST),
2. Clinical and Laboratory Standards Institute (CLSI),
3. British Society for Antimicrobial Chemotherapy (BSAC) or
4. Société Française de Microbiologie standards,

depending on which standards were used in each country [23–27]. Minimal inhibitory concentrations of antibiotics were not collected. Duplicate isolates (same species with same antibiogram from the same patient) identified within 4 weeks of the original isolate were excluded as part of the data collection protocol.

### Target bacteria

This study examined MDR patterns for three GNB, namely *Escherichia coli*, *Klebsiella pneumoniae* and *Pseudomonas aeruginosa*.

### Interpretation of reported antibiotic susceptibility

Individual antibiotics were grouped into antibiotic classes as defined by the MDR classification algorithms (Table 1) [11]. Isolates reported as I or R to an antibiotic representative of an antibiotic class were classified as non-susceptible to that class. In the case of AST results for multiple antibiotics representative of one class, the isolate was classified as non-susceptible if they were reported as I or R to any of the antibiotics tested from that class. Isolates were defined as MDR-GNB if they were non-susceptible to ≥3 relevant antibiotic classes [11].

### Identification of MDR-GNB bacterial isolates

The proportion of isolates of each of the three species considered to show MDR was then calculated using three sets of antibiotic classes (Table 1):

1. ARPEC set: MDR status was defined by applying the MDR algorithm and based on information from all classes reported to ARPEC;
2. EARS-Net set: MDR status was defined by applying the MDR algorithm, but based solely on information for classes included in the EARS-Net protocol;
3. Routine set: MDR status was defined by applying the MDR algorithm, and based on antibiotic classes with a high level of reported results across all ARPEC laboratories. Classes were included in this set if AST information was available for at least 85% of isolates. The level of required reporting was chosen to reflect classes routinely tested for the bacteria of interest in the majority of laboratories.

As both the EARS-Net and routinely tested classes are subsets of the ARPEC classes, an isolate classified as MDR on the basis of either set was also considered to be MDR based on the ARPEC set.

### Evaluation of single PACCs

It was also assessed whether specific PACCs, suggested to be critical indicators of MDR by European, US and global professional and/or public health bodies (Table 1), could identify MDR-GNB as detected on the basis of all available data; that is, the ARPEC set [7, 13–15].

The specific PACCs of interest were *E. coli* and higher-generation cephalosporins, fluoroquinolones, aminoglycosides and carbapenems, *K. pneumoniae* and higher-generation cephalosporins and carbapenems, and *P. aeruginosa* and carbapenems.

We defined its sensitivity as the proportion of isolates classified as susceptible for each PACC among those flagged as MDR from the ARPEC set, and its specificity as the proportion of isolates classified as non-susceptible for each PACC that was identified as not MDR from the ARPEC set.

### Statistical analysis

All statistical analyses were carried out using Stata® v12.1, StataCorp, College Station, TX, USA. Whenever 95% confidence intervals (CIs) are given for proportions, these were calculated by applying an exact method for binomial data.

## Results

In total, 685 isolates were included in the analysis (375 *E. coli*, 176 *K. pneumoniae*, 134 *P. aeruginosa*).

### Antibiotic classes included in the Routine set

The classes with reported AST results for the participating centres were very diverse, and there was no consistent pattern of classes among hospitals located in the same geographical region (data not shown). No laboratory consistently reported on all classes that were included in the ARPEC protocol. There was more consistency for the subset of EARS-Net antibiotic classes, with AST results available for at least 85% of isolates of all three species.

There were several classes for which AST data were also available for at least 85% of isolates. The additional frequently tested PACCs included *E. coli* and *K. pneumoniae* AST results for penicillins/beta-lactamase inhibitor (91 and 96% of isolates), folate pathway inhibitors (86 and 86%) and antipseudomonal penicillins/beta-lactamase inhibitor (85 and 85%). These were then included in the Routine set (Table 1). The only additional ARPEC antibiotic class relevant for *P. aeruginosa* MDR classification was monobactams, for which AST results were reported for only 47% of isolates.

### Identification of MDR status according to the EARS-Net, Routine and ARPEC sets

**Fig. 1:**
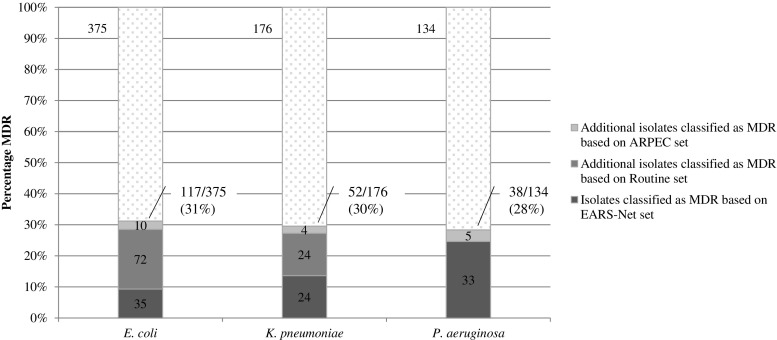
Number and percentage of isolates classified as MDR based on different sets of antibiotic classes (see Table 1 for definitions of the sets). The total number of isolates for each bacterial species is shown at the top of each bar

The proportion of MDR isolates based on the most complete ARPEC set was 30% (95% CI 27–34%) for all three GNB. Figure 1 shows the number of isolates classified as MDR using the EARS-Net set, the Routine set and the ARPEC set, and the overall proportion estimated as MDR for each pathogen.

Table 2 shows the proportion estimated as MDR for each set. Extending the set from the limited EARS-Net set to the Routine set identified an additional 96 MDR isolates, more than doubling the estimate of MDR-GNB from 13% (95% CI 11–16%) to 27% (95% CI 24–31%). This was most marked for *E. coli* and *K. pneumoniae* isolates (Fig. 1 and Table 2). A similar underestimation on the basis of the EARS-Net set was not observed for *P. aeruginosa*.

**Table 2:** MDR-GNB percentages based on the EARS-Net, Routine and ARPEC sets (see Table 1 for definitions of the sets)

	Total no. of isolates	MDR isolates		
		% MDR based on EARS-Net set (95% CI)	% MDR based on Routine set (95% CI)	% MDR based on full ARPEC set (95% CI)
*E. coli*	375	9.3 (6.6–12.7)	28.5 (24.0–33.4)	31.2 (26.5–36.2)
*K. pneumoniae*	176	13.6 (8.9–19.6)	27.3 (20.8–34.5)	29.6 (22.9–36.9)
*P. aeruginosa*	134	24.6 (17.6–32.8)	n/a	28.4 (20.9–36.8)
All GNB	685	13.4 (11.0–16.2)	27.4 (24.1–31.0)	30.2 (26.8–33.8)

For *E. coli* and *K. pneumoniae*, extending assessment to the Routine set meant that their MDR classification was based on three additional antibiotic classes (Table 1). The Routine set-based MDR status performed much better than categorisation based on the EARS-Net set alone. In contrast, comparing the Routine and ARPEC sets’ MDR status, only very few additional isolates were identified as MDR when the more complete ARPEC set was used.

### Identification of MDR status based on specific pathogen–drug combinations

The specific PACCs of interest were *E. coli* and higher-generation cephalosporins, fluoroquinolones, aminoglycosides and carbapenems (reported for 98, 99, 98 and 97% of isolates, respectively), *K. pneumoniae* and higher-generation cephalosporins and carbapenems (reported for 99 and 99% of isolates, respectively), and *P. aeruginosa* and carbapenems (reported for 98% of isolates).

*Escherichia coli* had the following PACC non-susceptibility profiles based on reported AST results: 13% (95% CI 9–16%) for third- and fourth-generation cephalosporins, 13% (95% CI 10–18%) for fluoroquinolones, 13% (95% CI 10–17%) for aminoglycosides and <1% (95% CI 0.1–2%) for carbapenems. For *K. pneumoniae*, resistance percentages for third- and fourth-generation cephalosporins were 32% (95% CI 25–40%) and for carbapenems 6% (95% CI 3–11%). *Pseudomonas aeruginosa* isolates showed 30% antipseudomonal cephalosporin resistance (95% CI 22–38%) and 31% carbapenem resistance (95% CI 24–40%). Resistance to higher-generation cephalosporins was 21% (95% CI 18–24%) for all three species. The corresponding resistance percentage for carbapenems was 8% (95% CI 6–11%).

**Fig. 2:**
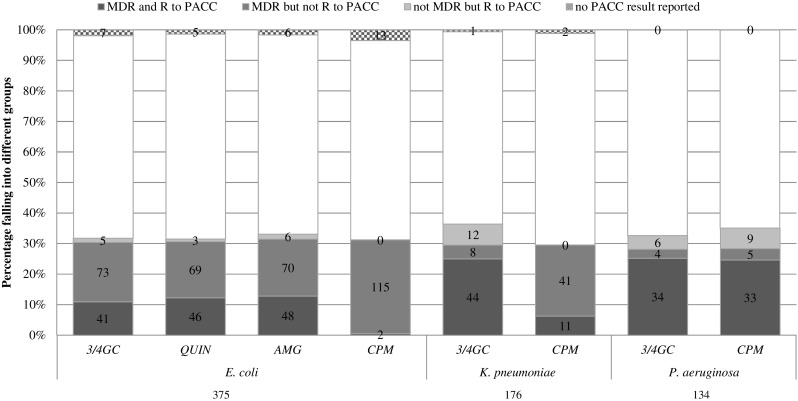
Number and percentage of isolates identified correctly or incorrectly as MDR based on individual pathogen–antibiotic class combinations (PACCs). The *white stacks* correspond to isolates neither resistant to the PACC nor identified as MDR on the basis of the ARPEC set (see Table 1 for definitions). The total number of isolates for each bacterial species are shown underneath. *3/4GC* third- or fourth-generation cephalosporin, *QUIN* fluoroquinolone, *AMG* aminoglycoside, *CPM* carbapenem. For *P. aeruginosa*, only cephalosporins with antipseudomonal activity were considered

Figure 2 displays the number and percentage of isolates that would be appropriately classified as MDR for each PACC. Isolates are classified as MDR on the basis of the ARPEC set.

For *E. coli*, resistance to the specified PACCs failed to correctly identify MDR status for more than half of the isolates. Aminoglycosides had the best sensitivity (i.e. ability to identify MDR when it was present) of 41% (Table 3). *Escherichia coli* carbapenem resistance was very rare in the ARPEC dataset, in contrast to MDR *E. coli*, and was of very little value in identifying MDR *E. coli*.

**Table 3:** Detection of MDR-GNB when specific PACC antimicrobial susceptibility testing results are assumed to represent MDR status. The percentage of isolates misclassified as MDR or not MDR based on PACC results is compared with MDR based on all ARPEC antibiotic categories (see Table 1)

		MDR classification			
		No. of MDR correctly identified	Sensitivity of PACC in % (95% CI)	No. of not MDR correctly identified	Specificity of PACC in % (95% CI)
*E. coli*	Third- or fourth-generation cephalosporins	41/114	36.0 (27.2–45.5)	254/259	98.1 (95.6–99.4)
	Fluoroquinolones	46/115	40.0 (31.0–49.6)	255/258	98.8 (96.6–99.8)
	Aminoglycosides	48/116	41.4 (32.3–50.9)	253/259	97.7 (95.0–99.1)
	Carbapenems	2/117	1.7 (0.2–6.0)	245/245	100.0 (98.5–100.0)
*K. pneumoniae*	Third- or fourth-generation cephalosporins	44/52	84.6 (71.9–93.1)	123/135	91.1 (85.0–95.3)
	Carbapenems	11/52	21.2 (11.1–34.7)	122/122	100.0 (97.0–100.0)
*P. aeruginosa*	Antipseudomonal cephalosporins	34/38	89.5 (75.2–97.1)	96/102	94.1 (87.6–97.8)
	Carbapenems	33/38	86.8 (71.9–95.6)	96/105	91.4 (84.4–96.0)

For *K. pneumoniae*, both cephalosporin and carbapenem resistance were more strongly associated with MDR status than for *E. coli* isolates. Third- or fourth-generation cephalosporin resistance had a sensitivity of 85%. However, again, carbapenem resistance was not predictive of MDR *K. pneumoniae* (sensitivity 21%).

For *P. aeruginosa*, both cephalosporin and carbapenem resistance showed a sensitivity of more than 85% for detecting MDR isolates. For all three GNB, the specificity (the ability to exclude MDR when it was absent) of the selected pathogen–drug combinations was above 90%. Thus, the rate of false classification of isolates as not MDR based on the absence of resistance to the PACCs reviewed was low.

## Discussion

The surveillance definition of MDR requires the availability of a large number of susceptibility testing results for the correct classification of isolates [11]. If monitoring and comparison of the prevalence of MDR-GNB is to be an aim for on-going surveillance activities collecting routine microbiology AST data, the optimal strategy for detecting MDR organisms from such data needs to be established. Current surveillance activities tend to request the AST results for a limited subset of antibiotic classes listed by the expert MDR classification algorithm [12].

In our dataset, the percentage of MDR-GNB isolates was significantly lower (13%) when based on a more limited set of antibiotic classes, such as that used by EARS-Net, compared with the full set available (30%). Utilising the full set of antibiotic classes reportable as part of the ARPEC project, the proportion of paediatric MDR *E. coli*, *K. pneumoniae* and *P. aeruginosa* isolates was around 30% and similar for all three pathogens. Such high levels of isolates with resistance to multiple drugs are concerning and of interest for tracking the epidemiology of resistant GNB over time.

Our study raises several important points regarding the potential of capturing MDR-GNB based on currently available routine microbiology data purely for surveillance:

1. Routine reporting of AST data by the 19 European laboratories participating in ARPEC only variably included results for requested antibiotic classes that are part of the classification algorithms for *E. coli*, *K. pneumoniae* and *P. aeruginosa*. A direct application of the MDR algorithms is, therefore, not possible.
2. Limited AST result data also cannot be used to reliably estimate the proportion of MDR-GNB. As the ARPEC dataset includes only European isolates, the performance of the current European surveillance system was evaluated. The EARS-Net set of antibiotic classes appeared to lack sensitivity for detecting MDR-GNB. Inclusion of additional frequently tested and reported antibiotic classes increased the detection of MDR *E. coli* and *K. pneumoniae* (from 30% detected by the EARS-Net set to 90% based on the Routine set for *E. coli* and from 46 to 92% for *K. pneumoniae*). This was in contrast to *P. aeruginosa*, for which the ARPEC set included only one additional antibiotic class compared with EARS-Net reporting.
3. A small number of individual PACCs currently represent the typical method for reporting antimicrobial resistance surveillance internationally. Disappointingly, resistance detected in individual PACCs was not reliable in detecting MDR isolates. This was especially marked for *E. coli* isolates, for which resistance to higher-generation cephalosporins, for example, had a sensitivity of only 36% for detecting MDR. *Escherichia coli* is the GNB with the largest number of antibiotic classes in the MDR classification algorithm and in ARPEC reporting. This may increase the detection of many different resistance combinations, especially if multiple different resistance phenotypes occur.

Some of the challenges may be explained by the fact that surveillance collects data primarily generated to inform clinical decision-making: approaches to AST are likely to be guided by the need to optimally inform patient therapy rather than by the need to generate a complete AST dataset for MDR classification. This type of selective AST based on clinical needs could introduce bias when these data are interpreted for public health purposes [28]. Bias could be magnified when laboratories engage in so-called first- and second-line testing: some antibiotic classes are evaluated only when resistance to antibiotics included in a first-line panel is detected [16].

Several limitations need to be considered when interpreting the ARPEC data. ARPEC does not cover all antibiotic classes recommended in the recent expert proposal [11]. It is, therefore, possible that some isolates identified as not MDR in ARPEC would, in fact, be MDR if AST data for all relevant classes were available. It is also possible that antibiotic classes tested for some of the reported isolates were suppressed during ARPEC data entry. This seems unlikely, given the relative uniformity of reporting for each species by each laboratory.

The actual percentages of MDR-GNB reported in this study should be interpreted with caution, as hospitals reporting to ARPEC were tertiary institutions with a patient population not representative of patients in other inpatient settings and potentially at higher risk of MDR-GNB [20, 21]. Pooling of data prohibits the identification of any differences between individual participating centres, some of which may have had higher or lower than average MDR-GNB percentages. Finally, the burden of MDR-GNB cannot be estimated because data are presented as resistance percentages rather than infection prevalence or incidence [29].

All isolates represent neonatal or paediatric blood cultures. The antibiotics used to treat bloodstream infections in neonates and children may differ from treatment choices for adults. This could be reflected in the antibiotic classes selected for AST, potentially limiting the transferability of the results to isolates from adults. However, most laboratories process microbiological samples from both adult and childhood patients. It is unlikely that AST strategies will be relevantly different for neonatal and paediatric isolates in these settings.

Surveillance of antimicrobial resistance patterns and trends is necessary to target interventions to reduce the selection and spread of resistant bacteria, and often relies on routine samples collected as part of on-going clinical care. The limitations and biases associated with the use of routine microbiology data in surveillance have been widely discussed [8, 28, 29]. Resistance percentages of individual PACCs and the EARS-Net set currently in use in Europe do not, on the whole, provide reliable MDR estimates. This study shows that, if MDR surveillance is to be added to the task list of on-going international surveillance, interpretation of the new algorithm will be limited by the variability in AST strategies in microbiological laboratories. MDR-GNB detection could be immediately improved by added surveillance of antibiotic classes already widely tested as part of clinical care. As demonstrated, a larger percentage of MDR-GNB isolates is likely to be identified with such an approach.
